# Correction: The Relative Contribution of Metabolic and Structural Abnormalities to Diastolic Dysfunction in Obesity

**DOI:** 10.1038/s41366-019-0404-2

**Published:** 2019-06-21

**Authors:** J. J. Rayner, R. Banerjee, C. J. Holloway, A. J. M. Lewis, M. A. Peterzan, J. M. Francis, S. Neubauer, O. J. Rider

**Affiliations:** Oxford Centre for Clinical Magnetic Resonance Research, University of Oxford, Oxford, UK

**Correction to: International Journal of Obesity**

10.1038/ijo.2017.239

This Article was originally published under a CC BY NC-ND 4.0 license, but has now been made available under a CC BY 4.0 license.

